# Assessing the efficacy of cash incentive policies in enhancing remittance inflows: Evidence from Bangladesh

**DOI:** 10.1371/journal.pone.0318342

**Published:** 2025-02-12

**Authors:** Muhammad Nafis Shahriar Farabi, Mahadee Al Mobin, Asir Newaz Khan

**Affiliations:** 1 Department of Economics, Bangladesh University of Professionals, Mirpur Cantonment, Dhaka, Bangladesh; 2 Bangladesh Institute of Governance and Management, University of Dhaka (Affiliated), Dhaka, Bangladesh; Bangladesh Agricultural University, BANGLADESH

## Abstract

The Government of Bangladesh (GoB) first implemented the cash incentive of 2 percent in July 2019 and continued the scheme with some modifications amid the pandemic to enhance remittance inflows through formal channels and ensure macroeconomic stability in the country. This study examines the impact of the cash incentive introduced by the GoB to boost remittance inflow using the Interrupted Time Series (ITS) analysis along with the Chow test for structural stability. While ITS analysis has been employed by numerous studies in the healthcare sector, but this paper uses such analysis for the first time in any type of migration study in Bangladesh. We have used ITS as it is most effective in measuring the impact of policy interventions that are expected to act either quickly after an intervention or within a stipulated time frame. The study is also the first to examine the region wise efficacy of policy intervention in the country. Monthly Remittance Inflow data from July 2013 to December 2021 has been used for the analysis. Chow test results conclude that the policy intervention had a significant impact while the ITS analysis findings demonstrated that the cash intervention significantly increased both aggregated and region-specific remittance inflows, highlighting the significance of the action. The overall findings revealed that the introduction of cash incentive in July 2019 resulted in an immediate, sustained increase of 6.68 percent in remittance inflows, with a further increase of 0.25 percent every month. Region wise analysis shows that the impact was highest in the USA & UK region and lowest in the Middle Eastern region, which signifies issues related to prevalence of hundi market, skillset of migrant workers, average monthly salary, and remittance sending costs. Our research provides policymakers with significant information to implement customized policies that ensure macroeconomic stability by enhancing remittance inflows through formal channels.

## Introduction

Over the last few decades, the labor migration sector and concerning issues and interests to migrant workers have become significantly crucial from the economic development perspective in Bangladesh. Similar to many developing countries and Least developed countries (LDCs), this sector has contributed largely in macroeconomic management, forex reserve, balance of payment (BoP) management, labor market dynamics, poverty alleviation, economic upliftment and overall economic development of the country.

Since 2000, remittances to low- and middle-income countries (LMICs), excluding China, have surpassed the volume of Official Development Assistance (ODA) [1]. Moreover, since 2015, remittances have become the largest source of external financing for these countries. Recent remittance figures show that it has surpassed the Foreign Direct Investment (FDI) flows to LMICs [2]. In Bangladesh, inward remittances are recorded to be significantly higher than Official Development Assistance (ODA), reaching 10.7 times the ODA volume in 2009, and although this ratio decreased, it remained substantial at 4.1 times in 2022 [2]. Globally Bangladesh is the 8th largest remittance recipient country and 6th largest migrant-sending country [3]. The country also receives a significant portion of the South Asian inward remittances.

An estimation of the total stock of international migrants worldwide put it at 272 million in 2019, of which international migrant workers made up 169 million accounting for almost 5 percent of the global labor force in host countries. A total of 146.9 million international migrant workers, which is about 87 percent of the total 169 million migrant workers reside in high-income and upper-middle-income countries [4]. South Asian migrant workers constitute a significant portion of the aforesaid country’s labor force. For instance, five Middle eastern countries make up the most prominent portion of residence for the Bangladeshi migrant workers. According to the Bureau of Manpower Employment and Training (BMET) data, these countries are Saudi Arabia, UAE, Kuwait, Qatar, and Oman, which accounted for 62.56 percent of the overseas employment for Bangladeshi migrants and 48.6 percent of total remittance earning for Bangladesh in 2023 [5]. Other regions that provided remittances include Southeast Asia in which Malaysia provides the highest proportion of remittances and the USA and Europe, with the UK being the European country to provide the most remittances.

**Fig 1:**
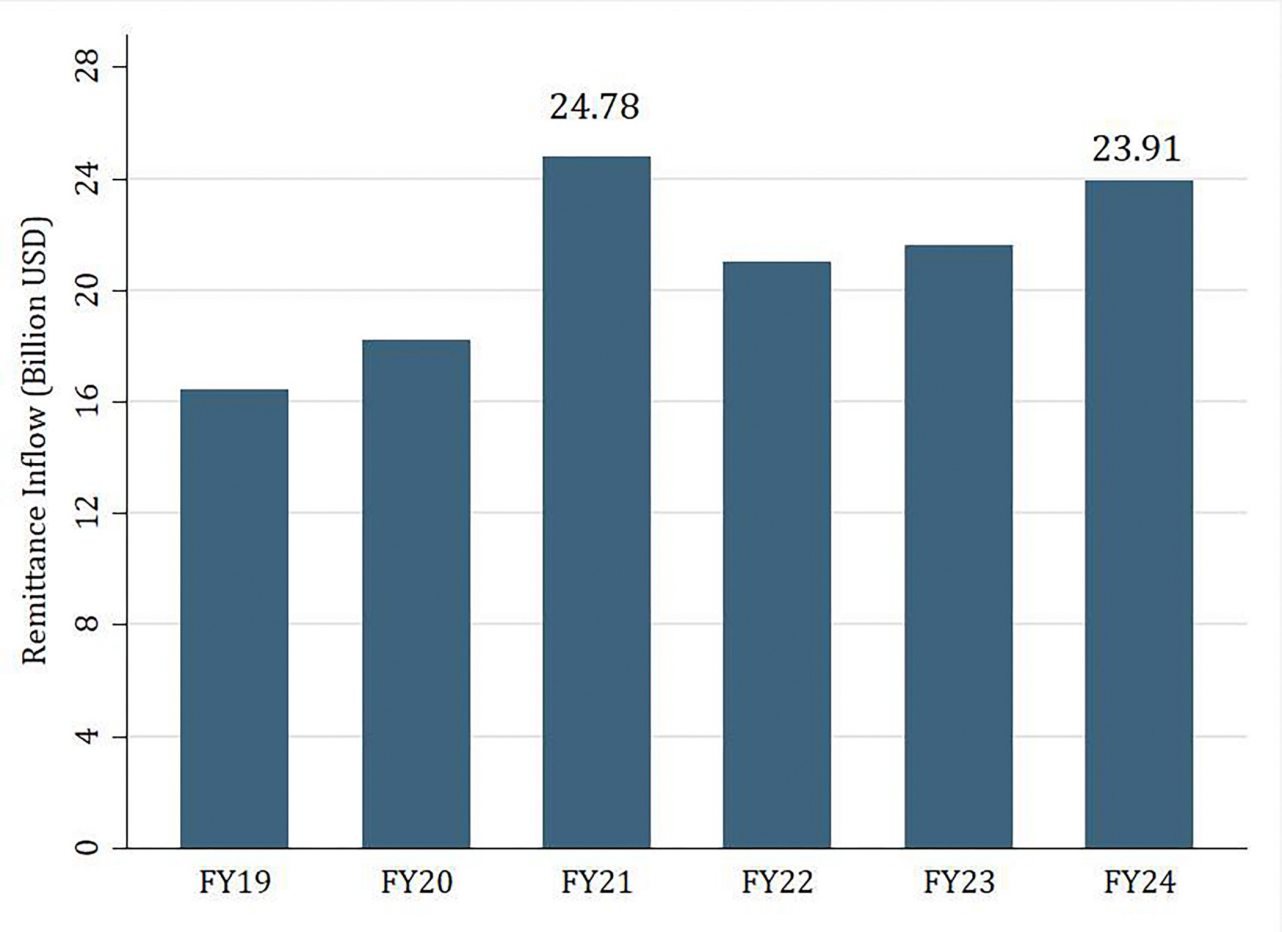
Total remittance earnings (FY19 -FY 24) [6].

**Fig 2:**
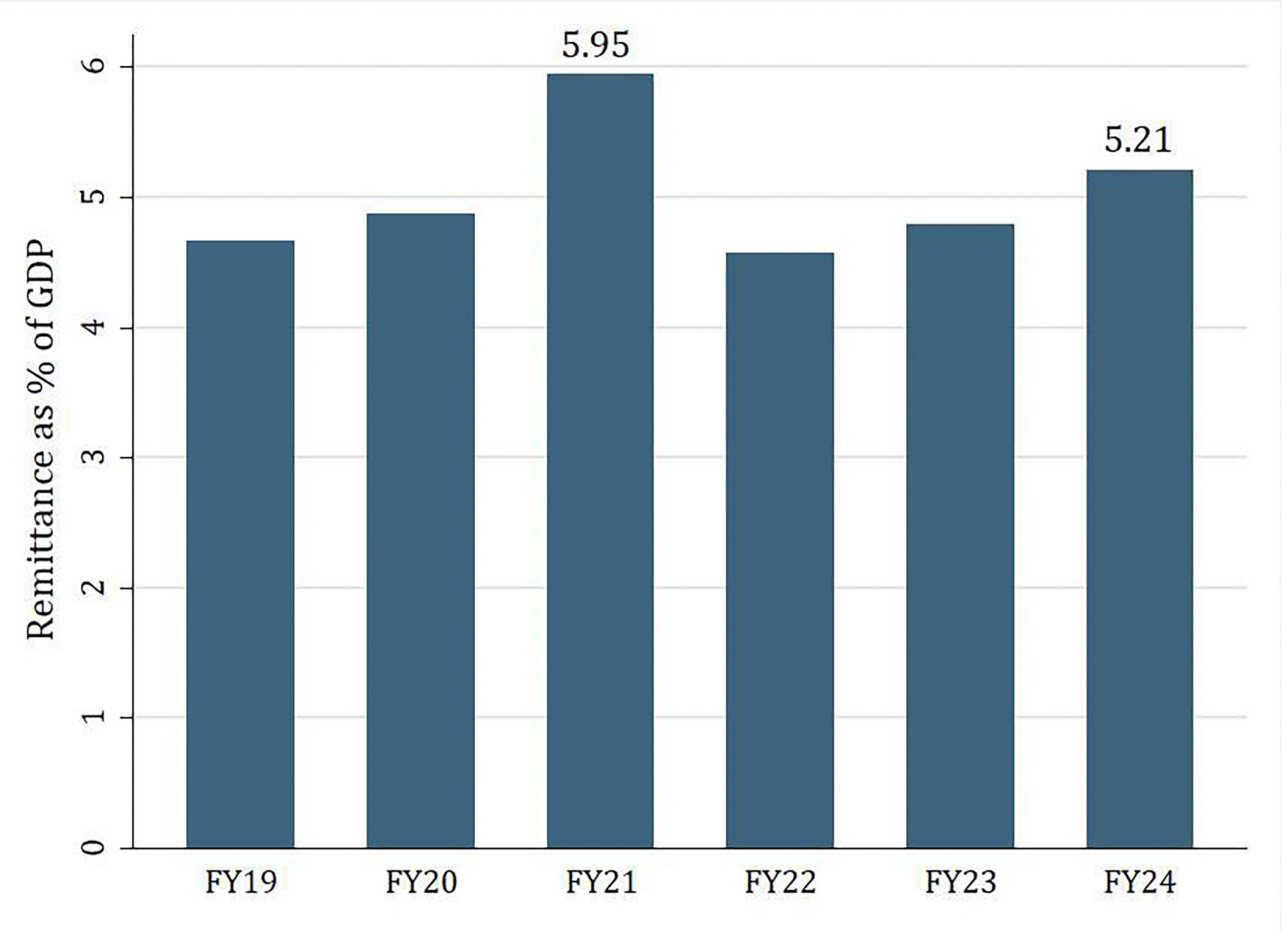
Remittance as % of GDP [6].

**Fig 3:**
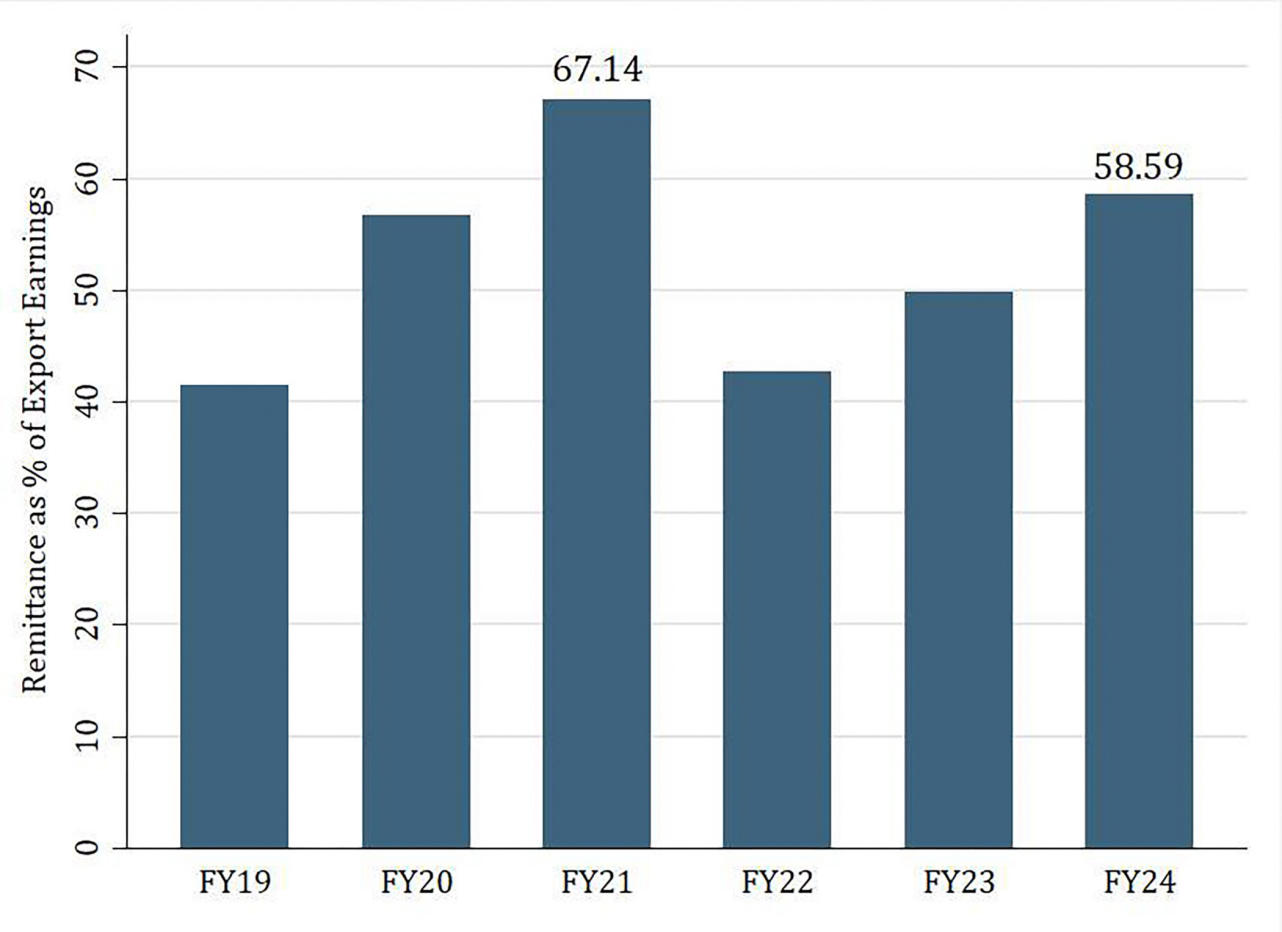
Remittance as % of export earnings [6].

As shown in Fig 1, Bangladesh exhibits an increasing trend in attracting inward remittances. The importance of remittances is highlighted in Figs 2 and 3; over the years, at roughly 5 percent, it makes a significant portion of Bangladesh’s GDP and more than 40 percent of the value of exports. In Fiscal Year (FY) 24 (July 2023-June 2024), remittances made up 5.21 percent of GDP and 58.59 percent of export earnings.

Remittances are transferred through both formal and informal channels. Formal channels are authorized systems of remitting money which include transactions through banks. These channels comply with the anti-money laundering policies and financial regulations of the country and ensure legal transfer of funds and contributes to the forex reserves. Formal remittances are defined as money transferred through the formal channels. However, oftentimes the inward remittances are bypassed through informal channels instead of formal channels due to issues such as high remittance transaction costs, strict documentary compliance, and limited financial infrastructure. Hundi is such an informal channel which is largely utilized by migrant workers of South Asia including Bangladesh [7]. This is considered an illegal and unlawful system in Bangladesh.

A significant portion of the inward remittances are transferred outside the official channels. The informal remittances are estimated to be 35–75 percent of the officially recorded figures in the developing countries [8]. In a recent study, the figure is reported as high as 30 percent of the total amount [9]. As this huge amount of remittance inflow remains unaccounted for, this affects monetary policy decisions and disrupts the channeling of remittances to productive sectors of the economy [8]. Remittance transfer through informal channels is also regarded as a threat to national security as it may also be used for illicit motives such as money laundering and terrorism financing.

Bangladesh Bank (BB), the central bank of Bangladesh introduced a 2 percent cash incentive for transfers up to USD 1500 without any verification which was effective from July 2019 [6]. The introduction of the remittance was justified on the grounds that it will encourage flow of additional remittances to the country and stimulate transfer of remittances from informal channels to formal channels. This incentive is applicable for the total amount of inward remittances received through formal channels. Remitters don’t have to apply to be eligible for the incentive scheme. When the remittance is sent through a formal channel, it is automatically credited and the money transfer operator (MTO) or bank handling the transaction applies the incentive to the recipient’s account in the country. Popular mobile financial services e.g. bKash has partnered with banks which has expanded the coverage in rural areas. BB reimburses banks for the incentive amount and supervises the process.

Following the COVID 19 global pandemic and the resulting lockdowns, several constraints were created to travel as well as income earning, hence there was a disproportionate impact on migrant workers and in turn on the remittances earned. The economies of the remittance recipient countries were particularly impacted; this led governments to implement policies that would seek to incentivize sending back remittances through a variety of means. In the context of Bangladesh, government continued the previously declared incentive scheme with some modifications. These included: (a) Increasing the ceiling for sending back remittances by individuals without supporting documents from USD 1500 (BDT150,000) to USD 5000 (BDT 500,000); and (b) Extending the amount of time to submit required documentation for individuals sending remittances more than USD 5000 (BDT 500,000) from 15 days to 2 months [10].

Against this backdrop, it is important to understand the effectiveness of the incentive scheme on remittance inflows as these are a significant source of income at the micro level for households as well as a key factor at the macro level as it provides a necessary source of foreign revenue to Bangladesh. The efficacy of the policy intervention also needs to be studied in terms of the flow of remittances from certain regions.

In studying GoB’s 2 percent incentive scheme, we seek to answer the following research questions in this study.

1. To what extent was the cash incentive effective in boosting total inward remittances?
2. How has the efficiency of the incentive scheme varied across different regions?

Studies are yet to examine the efficacy of cash incentives in boosting inward remittances in Bangladesh. This study contributes to existing literature in two important ways. First, it emphasizes on analyzing the efficacy of the cash incentive using a rigorous quantitative method named “Interrupted Time Series (ITS)”. In contrast, previous research has not used any quantitive method in this regard. Second, the study has analyzed the region-wise impact of such incentives which have not been explored before.

The paper is structured as follows. “Review of Literature” is divided into 3 parts. Firstly, literature on the impact of migrant remittances on macroeconomic and microeconomic variables has been discussed. Secondly, literature on the prevalence of informal channels of remittance sending and the underlying causes have been reviewed. Lastly, the paper reviews the studies conducted on the impact of policy interventions on inward remittance inflows. The “Methodology” part of the paper explains the methodology, econometric model, and empirical implementation of the study. The “Data” part of the study discusses the variables and data source. “Results and Discussion” presents the results derived from the econometric analysis and discusses possible reasoning. The “Policy Recommendations” part discusses the recommendations aligned with our study findings. Finally, “Concluding Remarks” draws an end to the paper.

## Review of literature

### Impact of migrant remittances on the macro and microeconomic scenario of an economy

Remittances that are sent to the countries of origin by the migrants working in the host countries significantly contribute to the development of micro as well as macro scenarios of these countries. For example, contributions coming in the form of remittances have a positive labor market impact (releasing pressure on domestic employment scenario) and income effect (development of the migrant households due to receiving remittances). Moreover, remittance inflow helps an economy in sustaining macroeconomic stability by having a positive impact on forex reserves, exchange rate stability, and import payment.

The neoclassical theory of migration states that due to wage differentials, migrants from low-wage countries migrate to high-wage countries [11]. [12] studies the impact of remittances on economic growth in South Asia. The study found a significant positive impact of inward remittance inflows on economic growth. The study results match the analysis of [13]. [14] studied the significance of remittances on economic development using the panel data for the period 1977–2017 for selected South Asian countries and found that inward remittances have a direct positive effect on GDP, household consumption & expenditure and a negative impact on inflation & govt. consumption expenditure. [15] analyzed if there exists a long-run relationship between remittances and economic growth. Using the panel data for the period 1977–2012 for four Asian countries (Bangladesh, India, Pakistan, and the Philippines), the study performed Pooled Mean Group (PMG) regression analysis. The results concluded that there exists a significant positive relationship among the studied variables. [16] found that inward remittances have a positive (negative) impact on economic growth in countries with high (low) efficiency of banks.

Inward remittances also have a significant positive effect on forex reserves. [17] analyzed the relationship between inward remittances and forex reserve for seven South Asian countries except Afghanistan. The study concluded that there is a positive and significant relationship between the studied variables for five of the aforesaid nations (except Sri Lanka and Pakistan). [18, 19] also in their study echoed the validity of the aforementioned relationship.

Poverty alleviation and employment are also positively affected by inward remittance. For the period 1993–2003, using the panel Gaussian Mixture Model (GMM) regressions for developing Asia and Pacific countries, [20] found that remittance inflows lead to higher household income and better employment opportunities which eventually lead to poverty reduction. The study findings resonate with the findings of [21, 22].

[23] analyzed the aforesaid relationship for 71 developing countries and concluded that inward remittances have a significant positive impact on the severity, level, and depth of poverty. [24] examined the impact of international remittances on poverty alleviation in Bangladesh and found that the level of poverty is significantly higher in households that don’t receive remittances and vice-versa. The paper also conducted the binary logistic regression model and concluded that if a household receives remittances, then the probability of that household being poor declines by 28.07 per cent. [25–27] also in their paper validated the aforesaid relationship.

Higher remittance inflows contribute to a rise in educational attainment [28–30]. Micro-level studies also show that inward remittances have a significant positive impact on the educational attainment of migrant households [31].

However, some authors argue that migration to the host countries leads to a loss of human capital which is referred as “brain drain”. This phenomena has a negative impact on the origin country’s economy [32].

### Preference for the informal channel in remitting money

One of the key hindrances to boosting remittances is the active presence of informal systems of remitting money such as “hundi”. Some of the key reasons for remittances being sent through informal channels are: (a) High remittance transfer costs (b) Lack of financial market infrastructure (c) Migrant’s characteristics.

Financial infrastructure, foreign exchange rates i.e. forex rates, and regulatory compliances result in high remittance transaction costs. There exists a significant difference between the interbank and hundi/hawala market, and kerb market exchange rate [8]. This difference between the exchange rates plays an instrumental role in convincing remitters to send money through informal channels. For example, there exists strong demand among the remitters to send money through informal channels in Sri Lanka as it offers more than 15 rupees than the interbank rate [33]. A study on labor migration by ILO shows that migrants sending USD 200 remittances through informal channels pay just 2 percent in fees while through formal channels such as banks, migrants paid 6.5 percent in fees [34]. A significant portion of the South Asian migrant workers are low-skilled and earns lower wages. For instance, according to the BMET, fewer and semi-skilled migrant workers constituted 50.5 percent of the total migrant workers in 2000 which had increased to 74.7 percent in 2010 and declined to 48.5 percent in 2019 [35]. As a result of the high proportion of low and semi-skilled migrant workers, they mostly remit small portions of money and don’t want to bear higher transaction costs [8]. Moreover, the remitters don’t want to face the hassles of formal documentation. Migrant workers lack language proficiency and tend to have lower literacy levels. This results in mistrust of formal financial systems and anxiousness among the migrant workers, diverting remittances to informal channels.

### Impact of policy interventions on inward remittances

Different studies have put light on the impact of policy intervention on inward remittance inflows. [36] studied the policy options related to attracting remittance inflows through formal channels. The study concluded that various policy interventions such as providing incentives, dismantling controls, and streamlining financial systems have contributed to the higher remittance inflow through formal channels in Nepal. [37] studied the influencing factors for higher inward remittances and found that reformation in India’s exchange rate policy was one of the important influencing factors, which influenced inward remittance inflows through formal channels. Liberalizing the exchange rate regime, the Indian govt. dis-incentivized the usage of informal channels to remit money. This resulted in higher inward remittance inflows through formal channels.

The Central Bank of Nigeria adopted a cash incentive policy named the “Naira 4 Dollar Scheme” in March 2021. Under this incentive policy, remitters sending money home through CBN’s INTERNATIONAL MONEY TRANSFER OPERATORS (IMTOs) received N5 for remitting every USD1. This cash incentive policy worked very well for the country. Weekly diaspora remittances on average exceeded USD 100 million in January 2022, which was only USD 6 million in December 2020 [38]. The Central Bank of Sri Lanka (CBSL) also came up with cash incentives of LKR 2 for each dollar remitted through formal channels in 2020. However, an upward trend was not observed in the inward remittance inflows. Inward remittance inflow was USD 4.12 bn in July-Aug 2020, which declined to USD 3.23 bn in Jan-June 2021. Remittance inflow further declined to USD 2.17bn in the July-Aug 2021 period [39, 40]. A shift to informal channels and mistrust of government has contributed to a decline in inward remittance inflows in the country [41].

[17] is one among the few literatures, which has studied the impact of the Bangladesh govt.’s cash incentive policy to encourage remitters to remit through formal channels. The study using simple descriptive statistics found that the cash incentive has a positive impact on remittance inflows in Bangladesh.

To our knowledge, there exists no quantitative paper, which has used any econometric model to figure out the impact of policy intervention such as cash incentives on inward remittances, particularly in the Bangladesh context. This study using an econometric model reports the efficacy of cash incentives on inward remittance inflows. Region-wise impacts have been also studied in this paper using the model, which is absent in the previous literatures.

## Materials and methods

### Data

#### Interrupted Time Series (ITS)

Remittance data has been taken from the Bangladesh Bank website for the ITS analysis. Monthly data on the studied variable has been compiled from July 2013 to December 2021. The intervention was declared in June 2019 and was effective from July 2019. For the pre-intervention period, this paper uses data from July 2013 to June 2019; and for the post-intervention period, data from July 2019 to December 2021 has been used. GoB announced an increased cash incentive of 2.5 per cent in January 2022. Hence, we have limited the study period to December 2021. The Middle Eastern region included five countries: Saudi Arabia, UAE, Qatar, Bahrain, and Oman while the Southeast Asian region includes Malaysia and Singapore. As the name suggests, the USA and UK region included the countries USA and UK.

#### Chow test

For carrying out the Chow Test, the sources of data are the Bangladesh Bank (BB), and Bureau of Manpower, Employment and Training (BMET). Monthly data on the studied variables have been compiled from July 2013 to December 2021.

### Methodology

In this study we use ITS analysis to study the impact of the intervention both in terms of immediate and long terms. We initially used chow test to check whether there exists a statistically significant impact of the policy intervention in the inward remittance inflow and finally we use ITS to quantify the immediate and sustained effect of the policy intervention.

#### Chow test

The Chow test is a commonly used method in econometrics to test for structural stability. It shows the presence of structural breaks in the model. If there exists such a break, the coefficients between the regression lines are found not to be equal. This test estimates a pooled model using the entire dataset and two separated models using Pre- and Post-Intervention data. The following formula is used to conduct the test using the chow’s ratio [42], which has evolved over the years:

$$F=\frac{({RSS}_{pooled}-({RSS}_{pre}+{RSS}_{post}))/k}{\left({RSS}_{pre}+{RSS}_{post})/(n_{pre}+n_{post}-2k\right)}$$

Here,

RSS_pooled_ is the residual sum of squares for the pooled model

RSS_pre_ is the residual sum of squares for the pre-intervention model

RSS_post_ is the residual sum of squares for the post-intervention model

k is the no. of parameters estimated in the respective models, which includes the intercept

n_pre_ is the no. of observations in the pre-intervention period

and n_post_ is the no. of observations in the post-intervention period

As the “unrestricted” model is run in two parts, (*n*_*pre*_+*n*_*post*_ -2k) is used in the denominator.

The variables studied in the Chow test are as follows:

Dependent Variable:

Total Remittance Inflows (TRI): This is the value of total remittances sent to the country by the Bangladeshi migrants living abroad.

Independent Variables:

Exchange Rate (ER): Depreciation of domestic currency leads to higher remittance inflows as they are earning more BDT on each dollar remitted.

General Inflation Rate (GIR): Domestic Inflation has an indirect positive relation with remittance inflow. Higher domestic inflation results in higher demand for money by the migrant’s relatives in the sending country. This translates into a higher remittance inflow.

Food Inflation (FI): Higher food inflation leads to higher remittance inflow. Food inflation denotes a rise in the price of clothing, housing, and another household spending.

Non-Food Inflation (NFI): Higher Non-Food Inflation leads to higher remittance inflow. Non-Food Inflation includes items such as rice, sugar, flour, meat, fish, and eggs.

Interest Rate of Schedule Banks on Advances (DR): This rate is the domestic interest rate. A higher domestic interest rate is favorable for investment. When the domestic interest rate is high, remittance inflows tend to be higher.

#### ITS

ITS analysis or "intervention analysis" is a dynamic tool of time series analysis that can be implemented given the following holds:

■ We possess data about an outcome across time (longitudinal data)
■ We want to comprehend how and if the outcome changed after an intervention, policy, or program was introduced for the entire population at a single point in time.
■ Our data must include observations from both before and after the intervention. The greater the number of observations on both sides of the intervention, the stronger your model will be (typically).

**Fig 4:**
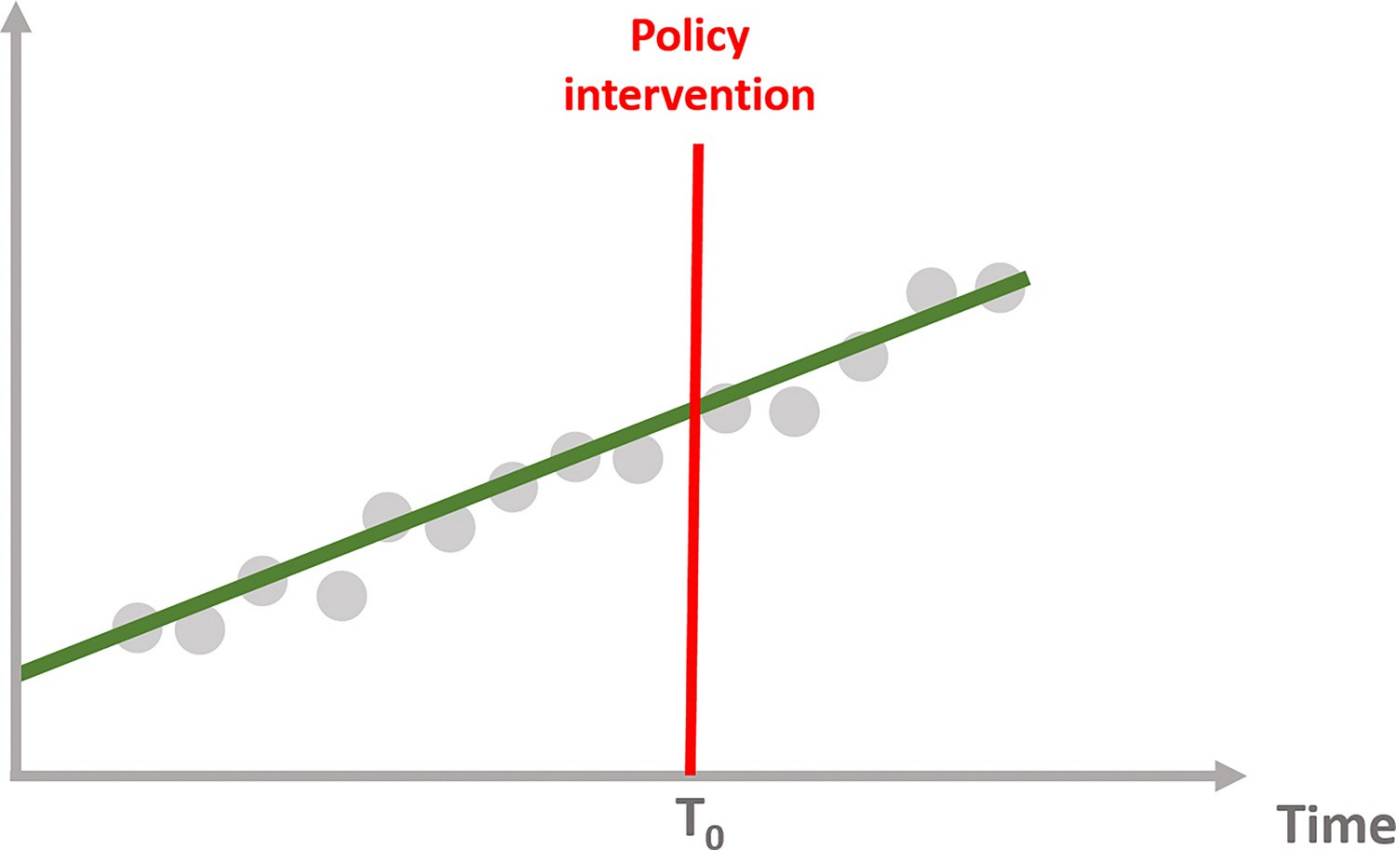
No effect of the intervention.

**Fig 5:**
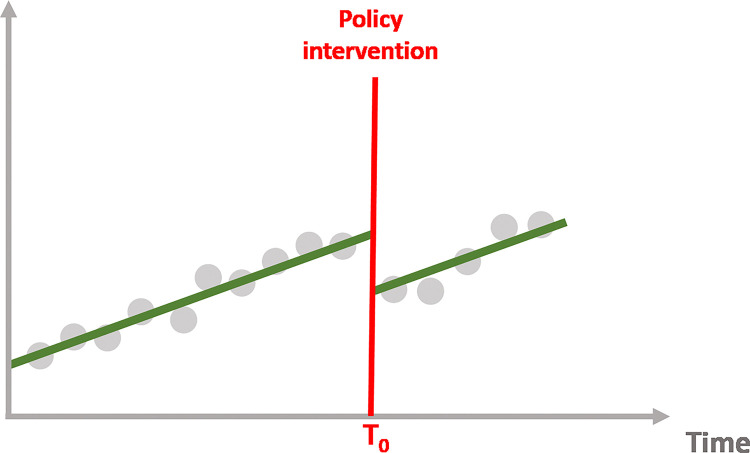
Immediate effect of the intervention.

**Fig 6:**
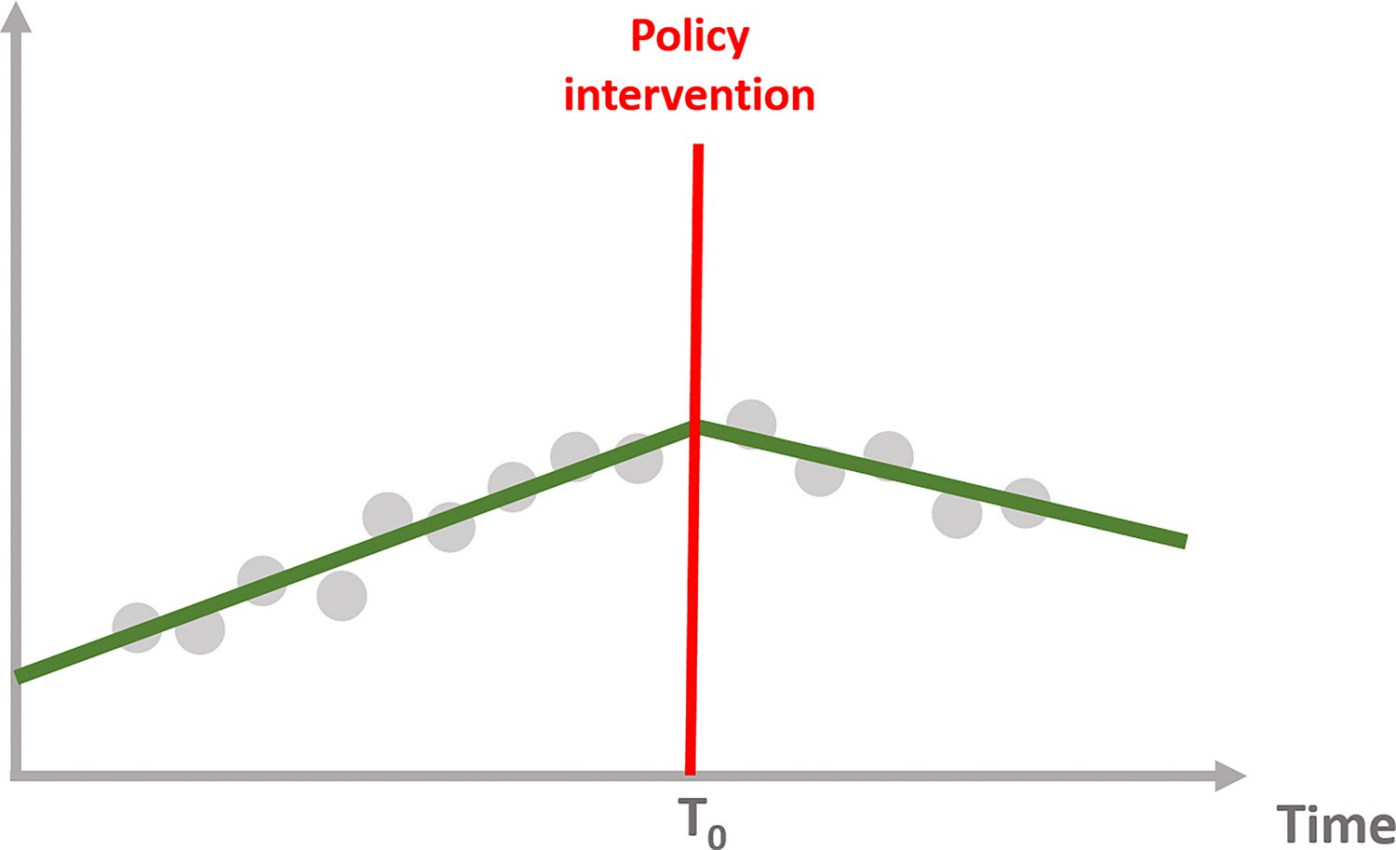
Sustained effect of the intervention.

**Fig 7:**
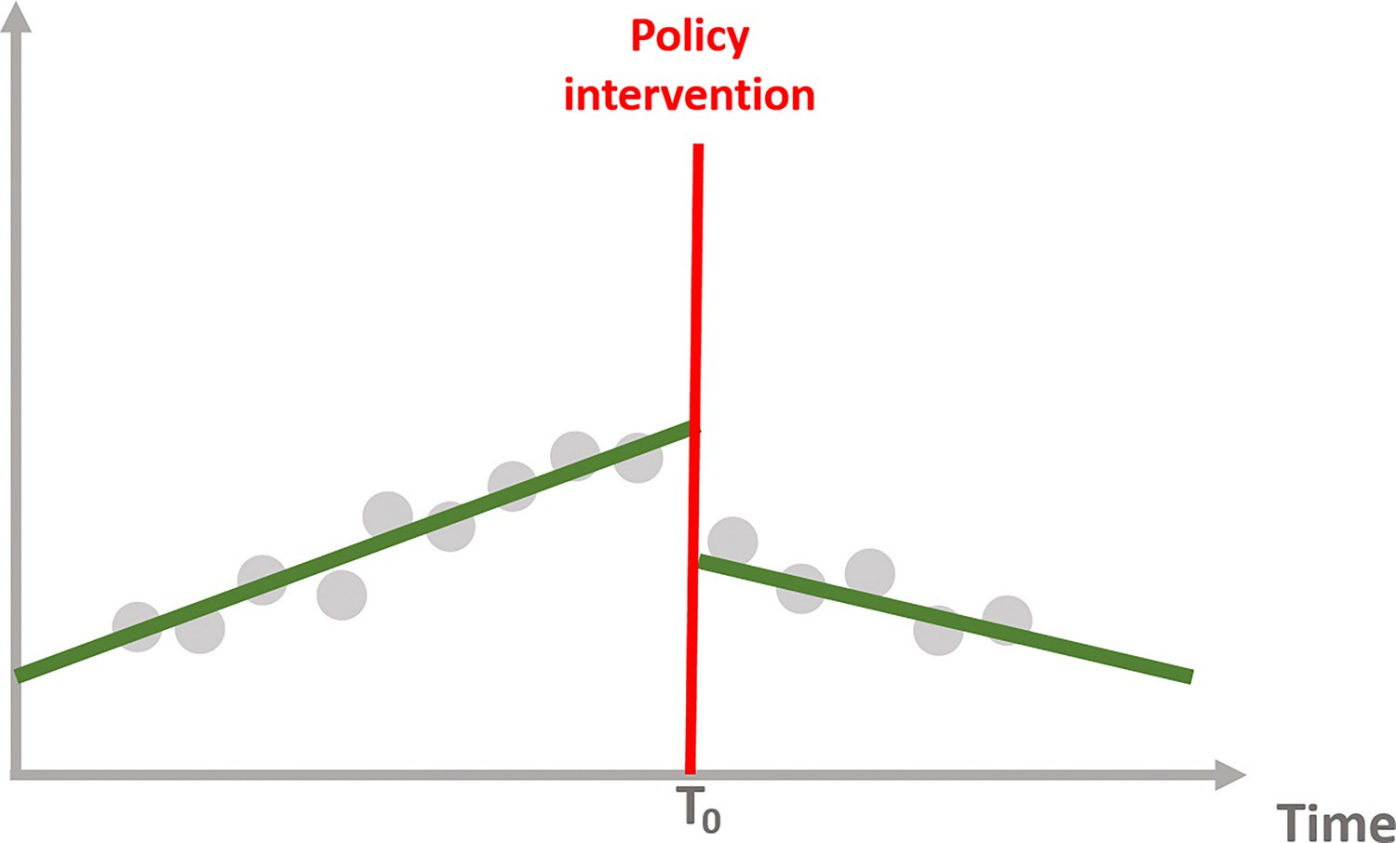
Immediate and sustained effect of the intervention.

Interrupted time series allows us to examine each effect, allowing us to determine whether the policy intervention has the following impact depicted on the Figs 4–7:

Typically, in order to enumerate the consequences of an intervention, pre and post analysis are deployed. These depend on the *"natural experiment"* produced by applying interferences, separating time into *"pre-intervention"* phases of *"post-intervention"*. Although, bias is more common in observational research based on small number of before and after intervention data. This is because of the fact that that these don’t factor into pre-existing underlying shorts and long-period tendencies [43]. In comparison, ITS longitudinally tracks the outcome prior to and following an intervention as a way to deal with these shortcomings. This makes the method more dynamic.

Whenever randomized controlled trials (RCTs) are not practicable and proper, ITS is regarded as one of the finest methods for proving causality [44, 45]. As a matter of fact, being paired with a control series, ITS designs frequently produce findings that are comparable to RCTs [46].

Multiple published articles [47–49] have explored the uses of ITS techniques to evaluate interventions. However, studies have mostly focused on segmented regression, the most straightforward kind of ITS analysis. Time is regarded as a regressor in segmented regression models; a fundamental segmented regression model may be written as follows:

$$Y_{t}=c_{0}+c_{1}\times time+c_{2}\times intervention+c_{3}\times time\;since\;intervention+\varepsilon_{t}$$

Where Y_t_ is the result for time point (t), the time variable implies the study time period, and the *intervention* classifies t in pre (0), post (1) intervention phase, and the *time since intervention* implies the time lapse since the intervention, with a value of 0 before the implementation of the intervention. A basic assumption of this genre of model is that errors (residuals) are white noise. However, time series frequently violate this premise.

In case of a time series having well behaved trends and distributed residuals that are independent, segmented regression is most applicable. In practice, data patterns can be obscure or difficult to recognize, with substantial fluctuation. Segmented regression may not be applicable for certain time series due to the challenges in representing the structure in autocorrelation. Autoregressive Integrated Moving Average (ARIMA) models are a substitute for segmented regression. The output of ARIMA models differs from segmented regression. Y_t_ regresses only on outcomes evaluated at the earlier time value (not on time itself). Although, the literature provides no direction on ways to adapt these class of time series models within the framework of ITS analysis. Due to the abundant availability of higher data volume and data intricacy for the purpose of research, ARIMA became a valuable tool for evaluators of large-scale treatments.

**Fig 8:**
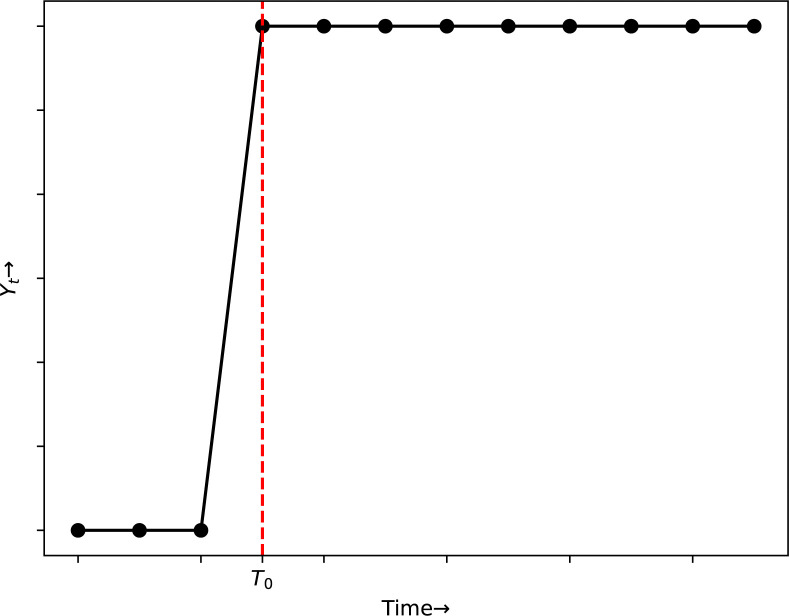
Plot of step function.


The objective of ITS analysis when applied to examine interventions is to quantify the "intervention effect," or the impact of the intervention’s execution on a given outcome. While there are numerous sorts of impacts that can be noticed, this article will focus on two of the most common: step change, and ramp which are as follows:

*Step change/ level shift*. Step change or level shift is a quick, persistent alteration in which the time series is transformed either up or down by a specified amount soon succeeding an intervention. The value of the level shift variable is 0 before the intervention and 1 subsequently as exhibited in Fig 8.

$$S_{t}=\{0;\;t<T_{0}1;t\geq T_{0}$$

Where, *T*_0_ is the time value of the intervention.

**Fig 9:**
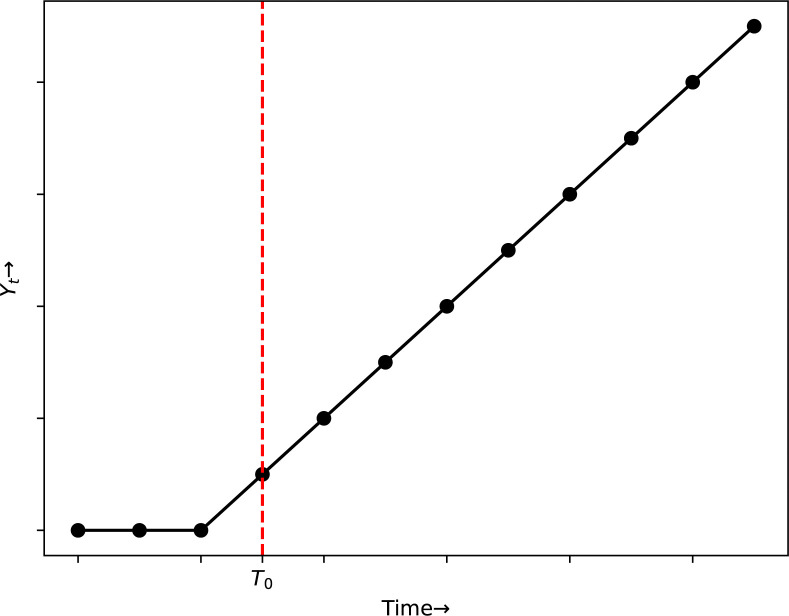
Plot of ramp function.


*Ramp*. A shift in slope shortly succeeding the intervention. It has a value of 0 before the intervention and a value increased by 1 at each step once the intervention begins as exhibited in Fig 9.

$$R_{t}=\{0;\;t<T_{0}t-T_{0}+1;t\geq T_{0}$$

Preferably, the probable impact shape of interference should be hypothesized beforehand. The form is determined by a number of variables, which includes the behavior of the intervention, such as if it is momentary, and the particular result is evaluated. For certain interventions, the variation is perfectly characterized using mix of impact variables, e.g., conventionally there would be both a level and a slope shift. We can use information criteirons to identify the best blend of impact components when there are several alternative models.

If changes could take place before the intervention’s implementation is also an important aspect to consider. Additionally, it is crucial to validate whether there is a change. To avoid erroneous associations, we recommend establishing beforehand a suitable period of time during which the effect is expected to be detected, based on subject knowledge or past study. The optimal delay among these alternatives can be found during the modeling phase [50].

In ITS analysis, ARIMA anticipates *Y*_*t*_ without the intervention i.e. the "counterfactual" and evaluates how the observed deviates from this prediction. In contrast to segmented regression, it is unnecessary to include time or seasonal dummy variables in the ARIMA model, as ARIMA through differencing can eliminate trends and seasonality. If differencing eliminates the trend, before and after intervention trends can not be predicted by the model.

Time can be introduced as a covariate and AR and MA terms can be integrated to account for autocorrelation if the estimation of the pre-and/or post-intervention slope is needed [51, 52].

This paper aims to use Seasonal Auto Regressive Moving Average with exogenous factors (SARIMAX) model.

## Results

### Chow test

The Chow test involves regression model for combined and separated data. The pooled model presents a single regression for the pre- and post- intervention combined data while the separated models present separate regression for the pre- and post- intervention period.

Pooled Model: TRI_t​ =_ β_0_​+β_1_​ER_t_​+ β_2_​GIR_t_ + β_3_FI_t_ + β_4_NFI_t_+ β_5_DR_t_ + ϵ_t​_

Pre-Intervention Model: TRI_t​_^(pre)^​ = β_0_^(pre)^​+β_1_^(pre)​^ ER_t_ ​+ β_2_^(pre)​^ GIR_t_+ β_3_^(pre)​^FI_t_ ​+ β_4_^(pre)​^NFI_t_ + β_5_^(pre)​^DR_t_ +ϵ_t_^(pre)​^

Post-Intervention Model: TRI_t​_^(post)^​ = β_0_^(post)^​+ β_1_^(post)​^ ER_t_ ​+ β_2_^(post)​^ GIR_t_+ β_3_^(post)​^FI_t_ ​+ β_4_^(post)​^NFI_t_ + β_5_^(post)​^DR_t_ +ϵ_t_^(post)​^

The null and alternative hypothesis are as follows:

H_0_: Slopes and the intercept of Pre-Cash Incentive scenario are not different from those of Post-Cash Incentive scenario (no structural breaks)

H_A_: Slopes and the intercept of Pre-Cash Incentive scenario differ from those of Post-Cash Incentive scenario

Table 1 shows the coefficients of the Pooled, Pre-Intervention, and Post-Intervention models for the Chow test. The post-intervention model exhibits a significant rise (21.54) in comparison to the pre-intervention (3.03***) and pooled (3.9***) models, suggesting a potential shift in baseline following the intervention.

**Table 1:** Coefficients of models for chow test.

	Models		
Coefficients	Pooled Model	Pre Intervention Model	Post Intervention Model
Intercept	3.9***	3.03***	21.54
Exchange Rate	0.03***	0.04***	-0.15
General Inflation Rate	1.73	-0.53	-6.25
Food Inflation	-0.95	0.34	3.84
Non-Food Inflation	-0.59	0.25	2.30
Interest Rate of Schedule Banks on Advances	-0.06**	0.03	-0.11**

Significance Code: ‘***’: p-value ≈0; ‘**’: p-value≤0.01

Here, Chow F-stat = 10.28, Calculated F, Fc = 2.20

As, Chow F-stat> Fc, we reject H_0_ and conclude that the slopes and intercept of the Pre-Cash Incentive scenario differ from those of the Post Cash Incentive scenario.

We can conclude from the chow test that there is a difference between the pre and post-cash incentive scenarios. This shows that the policy intervention had an impact on the inward remittance inflows. But, the test does not provide any information as regards the direction (positive or negative) or magnitude of the impact.

**Fig 10:**
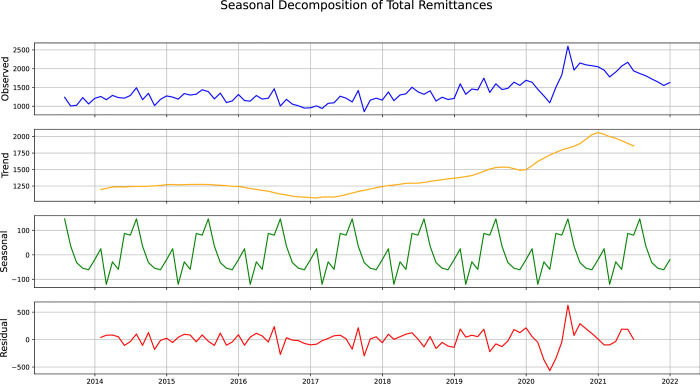
Seasonal decomposition of the total remittance (Worldwide).

Initially, we do a time series decomposition of the total remittance data using an additive model [53]. The result of this is observed in Fig 10. It is evident from the figure that the total remittance exhibits a yearly (12 months) periodicity. The same has been observed for the regional data, i.e., the Middle East, Southeast Asia, the US and the UK, which have been well portrayed in S1–S3 Figs, respectively. Thus, we will consider the periodicity of the data to be yearly, i.e., 12 months.

ITS is used extensively in health interventions starting from the introduction of vaccines to the mandate of using helmets. It has also been used to evaluate the impact of unanticipated issues like the global financial crisis. In this method, the pre and post-intervention period must be clearly defined. Short-term outcomes that are anticipated to change either reasonably rapidly after an intervention or after a defined period operate best with ITS [47].

### ITS

#### Final models

The best fit models are selected for the lowest AIC. Residuals and their corresponding cross-validation (Ljung box test) are included in robustness check subsection.

The model best fit for remittance inflow from **worldwide**:

$$(5,1,0)\times {\left(0,1,0\right)}_{12}$$

**Fig 11:**
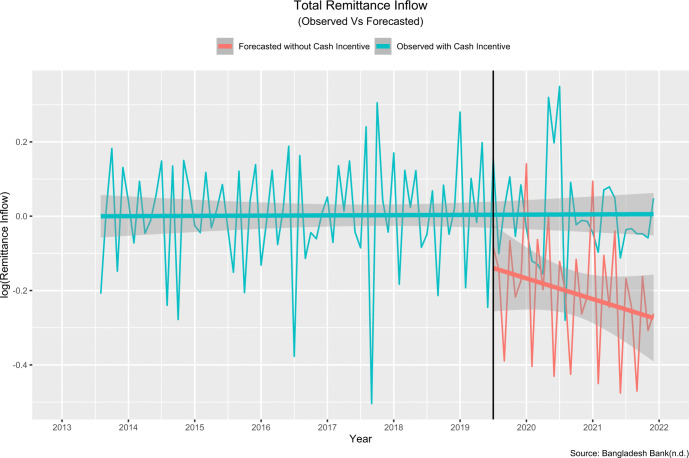
Observed and model predicted values in absence of the policy intervention (Worldwide/total).

The estimated change in slope was 0.25 percent, and the projected step change was 6.68 percent. This means that the introduction of cash incentive in July 2019 resulted in an immediate, sustained increase of 6.68 percent in remittance inflows, with a further increase of 0.25 percent every month as portraited in Fig 11. In other words, the remittance inflow increased by 6.93 (6.68+0.25) percent in July 2019 than the predicted remittance inflow had the intervention not been introduced. Fig 11 compares the values observed and predicted by the model in the absence of the intervention.

The model best fit for remittance inflow from the **Middle East:**

$$(5,1,1)\times {\left(1,1,1\right)}_{12}$$

**Fig 12:**
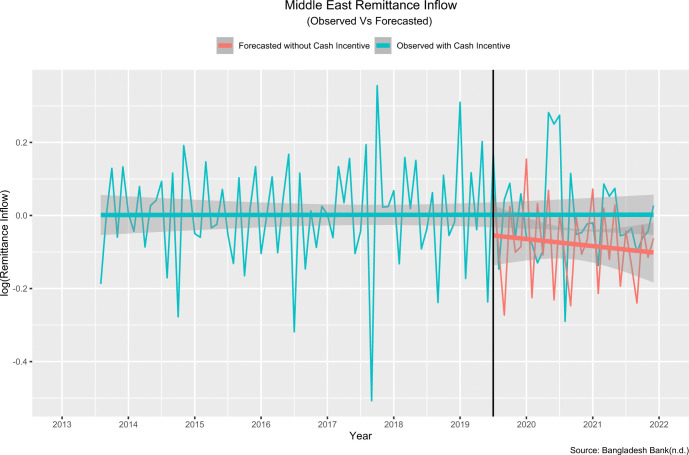
Observed and model predicted values in absence of the policy intervention (Middle east).

The estimated change in slope was 0.08 percent, and the projected step change was 4.40 percent. This means that the introduction of cash incentive in July 2019 resulted in an immediate, sustained increase of 4.4 percent in inward remittances, with a further increase of 0.08 percent every month. In other words, the remittance inflow increased by 4.48(4.4+0.08) percent in July 2019 than the predicted remittance inflow had the intervention not been introduced. Fig 12 compares the values observed and predicted by the model in the absence of the intervention.

The model best fit for remittance inflow from **Southeast Asia:**

$$(3,1,0)\times {\left(0,1,1\right)}_{12}$$

**Fig 13:**
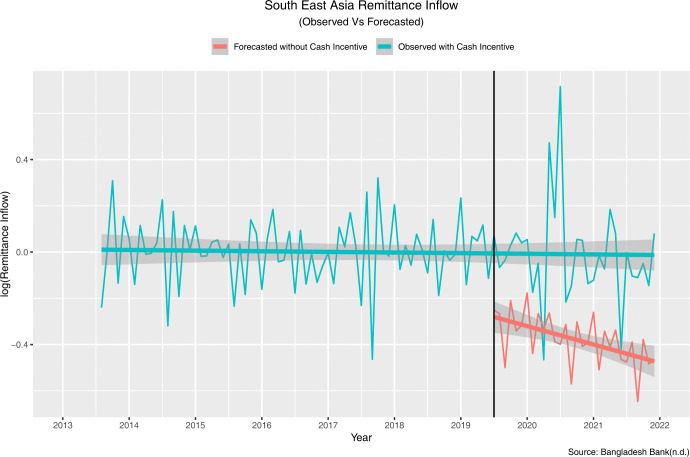
Observed and model predicted values in absence of the policy intervention (Southeast Asia).

The estimated change in slope was 0.75 percent, and the projected step change was 5.58 percent. This means that the introduction of cash incentive in July 2019 resulted in an immediate, sustained increase of 5.58 percent, with a further increase of 0.75 percent every month. In other words, the remittance inflow increased by 6.33 (5.58+0.75) percent in July 2019 than the predicted remittance inflow had the intervention not been introduced. Fig 13 compares the values observed and predicted by the model in the absence of the intervention.

The model best fit for remittance inflow from **USA and UK:**

$$(4,1,0)\times {\left(1,1,0\right)}_{12}$$

**Fig 14:**
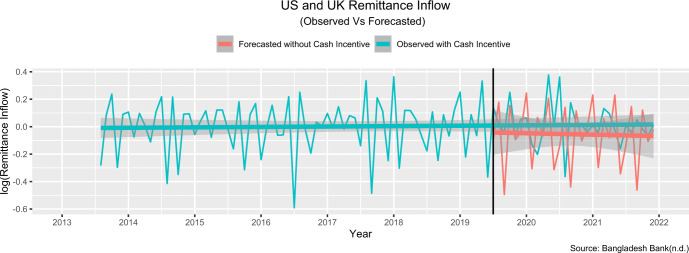
ARIMA model- predicted values and observed values in absence of the policy intervention (USA and UK).

The estimated change in slope was 0.06 percent, and the projected step change was 9.3 percent. This means that the introduction of cash incentive in July 2019 resulted in an immediate, sustained increase of 9.3 percent, with a further increase of 0.06 percent every month. In other words, the remittance inflow increased by 9.36 (9.3+0.06) percent in July 2019 than the predicted remittance inflow had the intervention not been introduced. Fig 14 compares the values observed and predicted by our ARIMA model in the absence of the intervention.

### Robustness check

To check for the robustness and the goodness of fit of the SARIMAX models, we will visually inspect whether the residuals are normally distributed with their mean of about zero, and to evaluate whether the model fits are good or not numerically, we shall resort to Ljung box test which tests whether a given time series especially residuals are autocorrelated. Its null and alternate hypotheses are as follows:

**H**_**0**_: The residuals do not exhibit significant autocorrelation/ The model is not a good fit.
**H**_**A**_: The residuals exhibit significant autocorrelation/ The model is a good fit.

#### Case 1: Total remittance inflow

**Fig 15:**
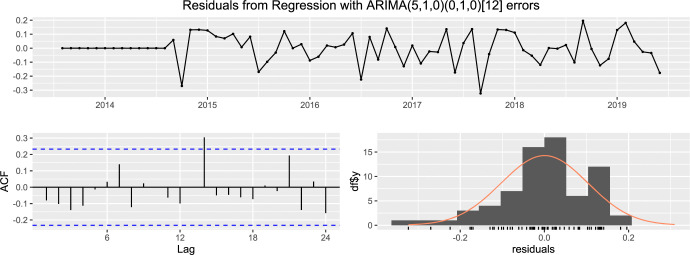
The residual of ARIMA (5,1,0) × (0,1,0)_12_ fitted with total remittance inflow.

In case of the worldwide remittance inflow, Fig 15 exhibits that the residuals do not exhibit any significant autocorrelations and residuals are normally distributed with its mean about zero.

#### Case 2: Middle east remittance inflow

**Fig 16:**
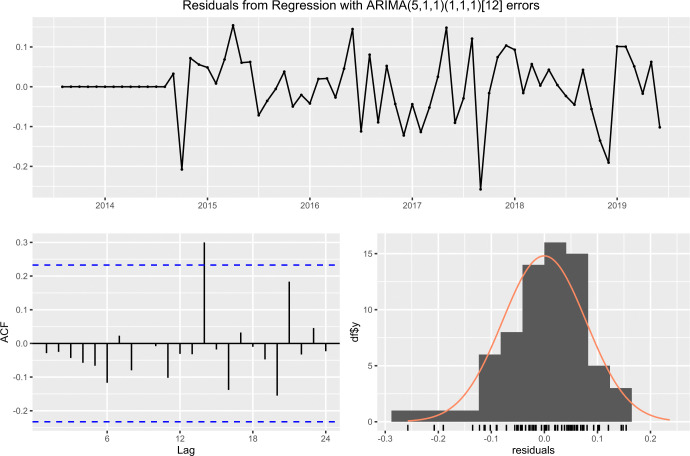
The residual of ARIMA (5,1,1) × (1,1,1)_12_ fitted with middle east remittance inflow.

In case of the middle east remittance inflow, Fig 16 exhibits that the residuals do not exhibit any significant autocorrelations and residuals are normally distributed with its mean about zero.

#### Case 3: Southeast asian remittance inflow

**Fig 17:**
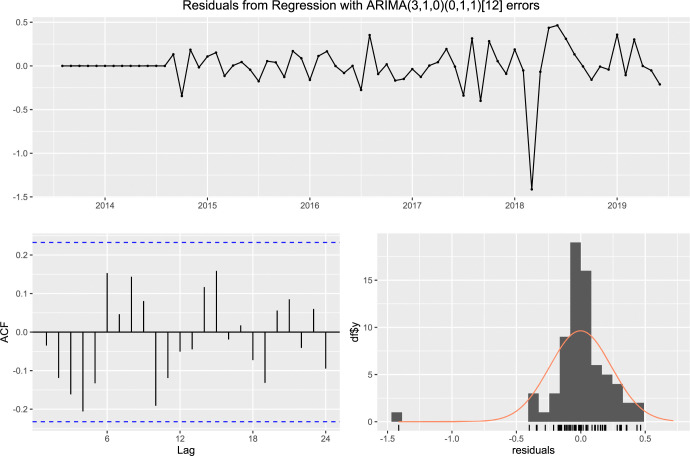
The residual of ARIMA (3,1,0) × (0,1,1)_12_ fitted with south-east Asian remittance inflow.

In case of the Southeast Asian remittance inflow, Fig 17 exhibits that the residuals do not exhibit any significant autocorrelations and residuals are normally distributed with its mean about zero.

#### Case 4: USA and UK remittance inflow

**Fig 18:**
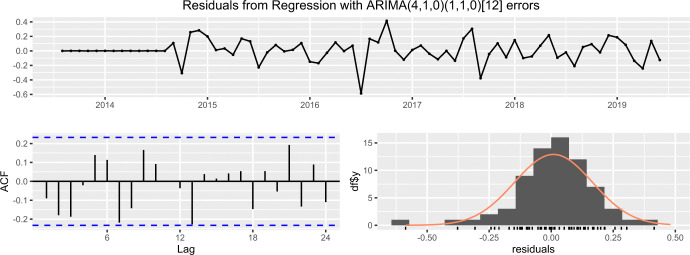
The residual of ARIMA (4,1,0) × (1,1,0)_12_ fitted with USA and UK remittance inflow.

In case of the USA and UK remittance inflow, Fig 18 exhibits that the residuals do not exhibit any significant autocorrelations and residuals are normally distributed with its mean about zero.

Now, statistically testing the goodness of fit for the fitted SARIMAX models, we use Ljung box test whose result for 5% level of significance has been interpreted in Table 2. Here, all the fitted models have non-autocorrelated residuals hence is a good fit for the data and we can consider the results from the model to be valid.

**Table 2:** Ljung box test interpretation.

Region	Best Fitted ARIMA Model	Ljung Box Test p value	Interpretation
**Worldwide**	(5,1,0) × (0,1,0)_12_	**0.06053**	**The model is a good fit.**
Middle East	(5,1,1) × (1,1,1)_12_	0.06762	The model is a good fit.
Southeast Asia	(3,1,0) × (0,1,1)_12_	0.05406	The model is a good fit.
USA & UK	(4,1,0) × (1,1,0)_12_	0.09925	The model is a good fit.

## Discussion

The findings of the study align with what one would expect from this type of policy intervention—if there is an informal (hundi/hawala) market in existence, migrant workers will generally be incentivized to shift from informal to formal market if and when the recipients back home receive additional amount (incentive) against the money they remit.

The Chow test of structural stability shows that there was a structural difference between the pre and post-intervention scenarios. The slopes and coefficients of the two scenarios differ. This shows that the policy intervention had an impact on inward remittances. But to check whether the impact was positive or negative, we have used the ITS method for further analysis.

Using the ITS analysis, we observe the sustained and immediate effect of policy intervention in attracting remittance inflows through formal channels. The immediate effect of the intervention is positive in the case of both total and region-wise remittance inflows. The immediate effect of policy intervention was highest in the case of the USA and UK region followed by the Southeast Asia region as depicted in Table 3. It shows that the migrant workers from the aforesaid regions responded to the cash incentive more than in the Middle East and other regions.

**Table 3:** Summary statistics of the impact of policy intervention in total and region-wise remittance inflows.

Region	Best Fitted ARIMA Model	Immediate Effect on inward remittance inflows (in percentage)	Sustained Effect on inward remittance inflows (in percentage)
**Worldwide**	(5,1,0) × (0,1,0)_12_	**6.68**	**0.25**
Middle East	(5,1,1) × (1,1,1)_12_	4.4	0.08
Southeast Asia	(3,1,0) × (0,1,1)_12_	5.58	0.75
USA & UK	(4,1,0) × (1,1,0)_12_	9.3	0.06

The impact of the intervention is found to be highest in the USA and UK region. The possible reason for such an impact is the skillset of the migrant workers. Bangladeshi migrants residing in the USA are generally skilled and skilled workers tend to earn higher wages and send more money to their home countries [54]. The Bangladeshi diaspora population in the USA has a higher median income (USD 54,000) for being highly educated and skilled. The aforesaid migrant workers also have a higher tendency to send money through formal banking channels, which may have resulted in increased inward remittances. The scenario is expected to be the same for the UK as per our findings.

In the Southeast Asia region, the significantly higher impact can also be attributed to the migrant worker’s higher average monthly salary. According to the BBS survey report of 2020, the average monthly salary for Bangladeshi migrants is BDT 46,895 and BDT 29,723 in Singapore and Malaysia respectively which is higher than Middle Eastern countries such as Saudi Arabia (26,180 BDT) and Oman (27,017 BDT). Signing of formal contracts between agencies and the migrant workers also play a key role in getting higher wages for such labor migrants. Migrant workers without any formal contracts are more prone to exploitation and receive lower wages. Two Southeastern countries, Singapore and Malaysia are two of the leading countries where Bangladeshi migrant workers are working with formal contracts while in Middle eastern countries like Saudi Arabia and Oman the figure is poor [55].

Skillset possessed by the labour migrants are a vital factor as regards the amount of remittances they send home. The migrant workers in the Middle East are less skilled and receive lower wages than Bangladeshi migrant workers in other regions [56]. This may cause a lower positive immediate effect on remittance inflows due to the intervention.

The lower positive impact in the Middle Eastern region may also be attributed to the fact that the Hundi/Hawala system is more prevalent in the Middle Eastern region [57]. “Hawala” means to transfer and has Arabic roots while “Hundi” has Sanskrit roots. In Bangladesh and Pakistan, the word “Hundi” is used while in India, Middle East and African countries, “Hawala” is used. These systems were considered safe and legitimate in the middle age but now they are considered neither legal nor safe. [7] also stated the same scenario in his paper. Remittance flows from the Middle East, particularly from Saudi Arabia and Emirates, have declined in spite of the very large number of migrant workers leaving for these countries in recent past years, which also shows probable diversion of remittance from formal to informal channels like hundi/hawala in this area [58].

Remittance sending costs also play a key role as regards the extent of impact generated by the policy intervention. A significant portion of the remittances from the Middle East comes from Saudi Arabia and UAE. But the remittance sending costs are high in these countries, especially it is unfavorable for low-income migrant workers. According to World Bank’s latest remittance price data, it costs a worker in UAE BDT 9.55 for remitting every BDT 100. From Saudi Arabia, it costs a migrant worker BDT 11.87 per every BDT 100 remitted [59].

In case of sustained effect, we see that the impact of cash incentive introduction had a sustaining effect on remittance inflow irrespective of the region but the degree of the impact varied across the regions. COVID-19 pandemic’s impact cannot be denied in this regard. As the pandemic started, migrant workers living abroad sent a significant amount of money to their home countries to help their families in distress. As uncertainties were looming, migrant workers in many cases sent all their savings. The borders were closed and the hundi or hawala system got disrupted which resulted in higher inward remittances through formal channels. As hundi/hawala system is more popular in the Middle East, we observe higher sustained effect in case of Middle East; higher than that of USA & UK.

In the case of Southaeast Asia region, the impact was highest. After the immediate effect, remittance inflow increased by 0.75 percent every month in the aforesaid region. The higher level of impact may have resulted due to various facts. For instance, migrant workers in Malaysia and Singapore received financial assisteance and aid packages that enabled them to send remittances back home in larger amounts and more frequently [60, 61]. Use of informal channels due to border closure and savings repatriation also contributed to this higher sustained effect.

Along with this continued cash incentive, relaxed regulations related to formal document submission have significantly facilitated remittance inflows, which has resulted in an overall significant sustained effect.

However, the 2.0 percent incentive was unlikely to ensure full transfer from informal channels to formal channels. Firstly, because for some remitters, the incentive may not be enough to offset the ‘incentive’ not to send money through formal channels (because the hundi/hawala margin is higher than the incentive amount). Accordingly, the impact is expected to be partial in terms of transfer from informal to formal channels. And secondly, there may be other forces at play that induce sending of money through informal channels (lower cost of sending money; easiness of sending money). Restrictions regarding use of forex exchange as a way to preserve forex reserves may also have led to higher transactions through hundi/hawala markets and resulted in less than expected efficacy of such policy interventions.

Hawala/Hundi system is also preferred by the money launderers to transfer funds. Those who would like to take money out of the country through informal hundi/hawla channel are ready to pay higher exchange rates since in majority of the cases these are illegally gotten money (black money) and such people are ready to pay a premium to get the money out of the country.

Although the aforesaid issues worked as a barrier to ensure a full-scale impact of the intervention, the study findings confirm that the policy intervention of introducing cash incentive has significantly attributed to sustained and immediate positive impact on inward remittances.

## Policy recommendations

Based on the study findings and challenges Bangladesh is facing, this study suggests the following policy recommendations which will be vital for boosting inward remittances in Bangladesh if implemented successfully.

### Continuation of the cash incentive

There exists a positive impact of policy interventions such as cash incentives on inward remittances as per our findings in this paper. Though region-wise the extent of the impact differs, the cash incentive certainly succeeded in attracting remittance inflow through formal channels. Continuation of the incentive program to attract more inward remittances will play a vital role in maintaining the macroeconomic stability of the country. GoB has increased the incentive in January 2022 to 2.5 per cent and in October 2023, an additional 2.5 percent additional incentive was decided to be provided by the banks [62].

The government has financed this cash incentive through budgetary support instead of printing money by the Bangladesh Bank. This was done to ensure macroeconomic stability without further worsening the inflationary situation in the country. However, this has to some extent limited fiscal space for which BB has created additional liquidity through purchasing treasury bonds and bills [63, 64]. As the cash incentive is crucial in attracting remittances, GoB should focus more on domestic resource mobilization to expand the fiscal space which in turn will not put pressure on the central bank.

Philippines, a major remittance recipient country uses indirect incentive mechanisms such as reduced remittance fees and tax-related incentives (e.g. tax-free investment accounts and savings programmes like the Pag-IBIG MP2 Savings Program) to encourage remittance transfer through formal channels without intensifying inflationary pressure [65–68]. These types of policies may also be considered in the context of Bangladesh.

Continuation and modification of cash incentive should be carried out along with implementing the following steps:

### Mitigate the difference between the official interbank exchange rate and hundi/hawala market rate

Due to the high difference between the exchange rates in official and hundi/hawala markets, remitters choose to send money through informal channels to benefit from the exchange rate differentials. There is also a significant difference in the kerb market as compared to the official exchange rate. The differential margin should be within BDT 2 to 3. On August 10, 2022, the interbank exchange rate was BDT 94.80 /USD while in the kerb market dollar was being sold at more than BDT 110. Hundi/Hawala market rate and kerb rate are different and the most suitable comparator of official rate is the hundi/hawala market rate. The stark differences observed in the kerb market should be addressed in this regard as the hundi/hawala system operates in line with the kerb market.

### Reduce the remittance sending cost

Remittance sending costs through formal channels play a key role in sending money through informal channels. As most of the Bangladeshi migrant workers are less skilled and mostly earn within the range of USD 200 to USD 300, higher fees on remitting money to Bangladesh works as a disincentive to send money through formal channels. As the amount of remittances increases, the remittance fee as a percentage of money remitted decreases which depict the law of “Economies of Scale”. According to the World Bank, there is enough scope to reduce the remittance sending fees by increasing competition in the remittance market. This will call for lowering the capital requirements for remittance services and allowing non-exclusive agreements with remittance agencies on the postal, banking, and retail networks could boost competition in the remittance sector [69].

Encouraging migrant workers in using digital remittance methods (e.g. PayPal, mobile money services, online banking transfers) rather than non-digital remittance methods (e.g. conventional bank transfers, in-person transfer systems of Western Union, MoneyGram, postal money orders) is also important. According to a World Bank report, compared to the 7 percent cost of the non-digital remittances, the cost was 5 percent in case of digital methods [1]. This highlights the significance of technological advancements in mitigating the financial burden on migrants.

### Minimize the migration cost

High migration cost has an indirect negative impact on remittance earnings of an economy and this is a common scenario in Bangladesh [70]. The migration cost in Southeast Asian countries such as Singapore and Malaysia exceeds BDT 5 lac and BDT 4 lac, respectively while in Middle Eastern countries such as Saudi Arabia and Qatar the migration costs also exceeds BDT 4 lac [55]. Strict measures needed to be undertaken to reduce this cost and introduction of affordable financing options should be made.

### Strengthen physical infrastructures

Physical infrastructure needs to be developed which will lead to affordable financial services for migrant workers. Physical infrastructure such as technology, internet, and electricity lead to greater financial inclusion and efficiency of the remittance transfer process and will decrease the cost of remittance transfer.

### Skilling up the migrant workers

The analysis reveals, the impact of policy intervention on remittance inflow is significantly lower in the Middle East compared to the USA and UK region. One of the possible reasons behind this is that Bangladeshi migrant workers residing in the Middle East are mostly unskilled and low-skilled. There has been a significant rise in demand for skilled migrant workers in Gulf countries, but Bangladesh has not been able to reap the benefits despite its large migrant population. Bangladesh govt. has planned to send abroad nearly 25 lac skilled new workers as stated in its 8th Five Year Plan (FYP). But as of FY22, the target was not met. The average monthly remittance sent by expatriates is only USD 203.33 while Chinese and Filipino migrant workers send an average of USD 564.10 and USD 395.71, respectively [71]. Skilled migrant workers tend to earn more than their unskilled counterparts. It is necessary for Bangladesh to skill up its potential migrant workers which will help the country to earn more remittances.

For skilling up the migrant workers, the Bangladesh government should strengthen the national expertise so that experts can perform need assessment & market analysis properly and further plan & execute necessary programs. Collaborative efforts with the foreign employers is also vital for enhancing the potential of the aspiring migrant workers. There should be also proper assessment and regular monitoring of developments as regards the skill development initiatives.

### Formalization and proper regulation of Hawala and other similar service providers (HOSSP)

Regulated HOSSPs are observed to report their activities to the central bank or other relevant financial authorities of the country. Licensed hawaladers also store more information about their customers while in case of unlicensed ones, only name and contact number are enough for a transaction [72]. If HOSSPs are formalized, properly regulated and supervised, they can be vital for financial inclusion and increased remittances & forex reserves in the economy.

### Re-continuation of the Pre-Shipment Inspection (PSI) program

The reduction of demand for the hundi market will lead to a decline in the importance of the market. To divert the remittances to formal channels, a mandatory PSI program should be re-continued as this will reduce the amount of under-invoicing. Bangladesh government as part of trade facilitation, has withdrawn the need for mandatory PSI [73, 74]. There should also be strict penalties introduced if any case of under-invoicing is found. This type of initiative will help to reduce the importance of the hundi market, and more money will be remitted through formal channels.

## Conclusion

This paper uses an econometric model to figure out the impact of cash incentives on inward remittances in Bangladesh which is unique in such types of studies. The Chow test for structural stability has been used to check if the intervention had a significant impact and the ITS analysis was used to figure out the direction and degree of the impact. The study findings revelaed that the policy intervention of the GoB was succeessful in boosting inward remittances both in the overall and regionwise scenario. The findings of the paper resonate the importance of policy interventions such as cash incentives for attracting higher remittance inflows through formal channels. The immediate impact was found to be highest in the USA & UK region while it was lowest in the Middle East. The sutained impact was highest in the Southeast Asia region. These variations can be attributed to issues concerning prevalence of hundi market, skillset of migrant workers, average monthly salary, and remittance sending costs.

Thus, the cash incentive policy intervention needs to be coupled with complementary policies such as reducing the remittance transfer cost, including more migrants and their households in the formal banking system, strengthening physical infrastructure, and skilling up the national experts and migrant workers.

While this paper focuses on the impact of cash incentives in attracting remittance inflows in Bangladesh, due to similar characteristics of the migration sector in South Asia, this paper is expected to contribute significantly to the migration policy choices for the South Asian countries. Furthermore, this study will have implications for remittance recipient countries worldwide.

The study has certain limitations. For instance, the ITS analysis didn’t consider the impact of COVID-19 pandemic in boosting inward remittances. As the intervention started in July 2019 and we considered the study period up to December 2021, the pandemic along with the cash incentive had also contributed to higher inward remittances through formal channels. Additionally, this study is limited to Bangladesh.

Further research may segregate the impact of the COVID-19 pandemic from the policy intervention and there could be separate study on other top remittance receiving countries who have introduced policies to enhance remittance inflows. There could be also separate studies on the efficacy of the 2.5 and 5 percent cash incentives introduced by the GoB. The study also shows the importance of conducing further research on region wise migrant worker’s skillset and how to improve the situation, which will eventually boost the inward remittance inflow.

## Supporting information

S1 Fig Seasonal decomposition of middle east remittance inflow. (EPS)

S2 Fig Seasonal decomposition of south-east Asia remittance inflow. (EPS)

S3 Fig Seasonal decomposition of USA and UK remittance inflow. (EPS)

S1 Dataset (CSV)

S2 Dataset (CSV)

S3 Dataset (CSV)

S4 Dataset (XLSX)
